# Effects of hypertonic saline versus mannitol in patients with traumatic brain injury in prehospital, emergency department, and intensive care unit settings: a systematic review and meta-analysis

**DOI:** 10.1186/s40560-020-00476-x

**Published:** 2020-08-12

**Authors:** Yukari Miyoshi, Yutaka Kondo, Hidetaka Suzuki, Tatsuma Fukuda, Hideto Yasuda, Shoji Yokobori, for the Japan Resuscitation Council (JRC) Neuroresuscitation Task Force and the Guidelines Editorial Committee, Yasuhiko Ajimi, Masaaki Iwase, Kyoko Unemoto, Junji Kumasawa, Jun Goto, Hitoshi Kobata, Atsushi Sawamura, Toru Hifumi, Eisei Hoshiyama, Mitsuru Honda, Yasuhiro Norisue, Shoji Matsumoto, Yasufumi Miyake, Takashi Moriya, Tomoaki Yatabe, Kazuma Yamakawa, Sunghoon Yang, Masahiro Wakasugi, Masao Nagayama, Kosaku Kinoshita, Hiroshi Nonogi

**Affiliations:** 1 Department of Emergency and Critical care Medicine, Juntendo University Urayasu Hospital, 2-1-1 Tomioka, Urayasu, Chiba, 279-0021 Japan; 2 Emergency and Critical Care Center, Japanese Red Cross Musashino Hospital, Musashino, Japan; 3 Department of Emergency and Critical Care Medicine, Graduate School of Medicine, University of the Ryukyus, Nishihara, Japan; 4 Department of Emergency and Critical Care Medicine, Jichi Medical University Saitama Medical Center, Saitama, Japan; 5 Department of Emergency and Critical Care Medicine, Nippon Medical School, Tokyo, Japan

**Keywords:** Traumatic brain injury, Hypertonic saline, Mannitol, Prognosis, Trauma

## Abstract

**Background:**

Intracranial pressure control has long been recognized as an important requirement for patients with severe traumatic brain injury. Hypertonic saline has drawn attention as an alternative to mannitol in this setting. The aim of this study was to assess the effects of hypertonic saline versus mannitol on clinical outcomes in patients with traumatic brain injury in prehospital, emergency department, and intensive care unit settings by systematically reviewing the literature and synthesizing the evidence from randomized controlled trials.

**Methods:**

We searched the MEDLINE database, the Cochrane Central Register of Controlled Trials, and the Igaku Chuo Zasshi (ICHUSHI) Web database with no date restrictions. We selected randomized controlled trials in which the clinical outcomes of adult patients with traumatic brain injury were compared between hypertonic saline and mannitol strategies. Two investigators independently screened the search results and conducted the data extraction. The primary outcome was all-cause mortality. The secondary outcomes were 90-day and 180-day mortality, good neurological outcomes, reduction in intracranial pressure, and serum sodium level. Random effects estimators with weights calculated by the inverse variance method were used to determine the pooled risk ratios.

**Results:**

A total of 125 patients from four randomized trials were included, and all the studies were conducted in the intensive care unit. Among 105 patients from three trials that evaluated the primary outcome, 50 patients were assigned to the hypertonic saline group and 55 patients were assigned to the mannitol group. During the observation period, death was observed for 16 patients in the hypertonic saline group (32.0%) and 21 patients in the mannitol group (38.2%). The risks were not significant between the two infusion strategies (pooled risk ratio, 0.82; 95% confidence interval, 0.49–1.37). There were also no significant differences between the two groups in the other secondary outcomes. However, the certainty of the evidence was rated very low for all outcomes.

**Conclusions:**

Our findings revealed no significant difference in the all-cause mortality rates between patients receiving hypertonic saline or mannitol to control intracranial pressure. Further investigation is warranted because we only included a limited number of studies

## Background

Intracranial pressure (ICP) control has long been recognized as an important requirement for patients with severe traumatic brain injury (TBI) [1]. Hypertonic solutions effectively reduce the patient’s ICP without brain perfusion impairment [2]. Although mannitol has been the recommended first-line osmotic agent in this setting for years, there are concerns that its use may lead to hypotension, especially in hypovolemic patients, as well as a rebound phenomenon with increased ICP, along with renal toxicity due to increases in serum osmolality [3, 4]. Thus, hypertonic saline (HS) has recently drawn attention as an alternative to mannitol and has been found to be more effective than mannitol for reducing ICP in TBI cases [5–7]. However, hypertonic saline is also associated with potential adverse effects, such as pontine myelinolysis [8]. Moreover, few clinical studies have focused on TBI-related outcomes, such as patient survival and long-term beneficial effects, and there is a lack of clarity regarding which HS is the most suitable for use in prehospital, emergency department, and intensive care unit (ICU) settings. Therefore, we aimed to assess the effects of HS versus mannitol strategies on TBI-related clinical outcomes.

## Material and methods

### Data sources and search strategies

The Japan Resuscitation Council (JRC) Neuroresuscitation Task Force and Guidelines Editorial Committee were established in 2020 by the Japan Society of Neuroemergencies and Critical Care, the Japanese Society of Intensive Care Medicine, and the Japan Society of Neurosurgical Emergency. The JRC Neuroresuscitation Task Force set clinically relevant questions for this systematic review.

To identify eligible trials, we searched the MEDLINE database via PubMed, the Cochrane Central Register of Controlled Trials, and the Igaku Chuo Zasshi (ICHUSHI) Web database [9]. The search was performed on October 1, 2019, and was not restricted by publication status, date of publication, or sample size, although only reports published in English and Japanese were included. The search terms were presented in Supplemental file 1. Systematic review and meta-analysis were conducted in accordance with the PRISMA guidelines [10], and was registered in the UMIN Clinical Trials Registry (ID UMIN000040184).

### Study selection

The titles and abstracts of the search results were retrieved from the databases. After the exclusion of duplicate studies, two investigators (YM and HS) independently screened the titles and abstracts for potential eligibility. In the case of disagreement between reviewers, the full-text report was used to determine study eligibility. Disagreements were resolved by consensus, although a third reviewer (TF) was consulted if consensus could not be reached. The full texts of potentially eligible articles were independently reviewed by two investigators (YM and HS), and a final decision regarding eligible studies was made after a discussion involving all authors and the resolution of disagreements by consensus.

We identified randomized controlled trials (RCT) for inclusion based on the research question and according to the PICO model (participants, interventions, comparisons and outcomes): participants, adults (≥15 years old) with TBI; interventions, administration of HS in prehospital, emergency department, and ICU settings; comparisons, administration of non-HS ICP-lowering agents in the same situation; and outcomes, the primary outcome was all-cause mortality.

### Data extraction

Data extraction was conducted independently by two investigators (YM and HS), with consensus used to resolve any disagreements. The extracted data included author, year of publication, country, study design, number of study participants, patient demographics, outcome measures, and inclusion and exclusion criteria.

### Study endpoints

We set all-cause mortality during the observation period as the primary outcome. The secondary outcomes were 90-day and 180-day mortality, good neurological outcomes, decline in ICP, and serum sodium level. According to the Grading of Recommendations Assessment, Development and Evaluation (GRADE), the primary outcome was defined a “critical” outcome and secondary outcomes as “important” or “critical” outcomes [11].

### Assessment of methodological quality: risk of bias assessment and GRADE approach

We adapted the Cochrane risk of bias tool to assess the quality of the included studies [12]. Each study was assessed for (1) random sequence generation (selection bias), (2) allocation concealment (selection bias), (3) blinding of participants and staff (performance bias), (4) blinding of related outcome assessments (detection bias), (5) true intention-to-treat analysis (attrition bias), (6) incomplete outcome data (attribution bias), (7) selective reporting (reporting bias), (8) early trial withdrawal bias, and (9) other sources of bias. We classified the studies as having a low, intermediate, or high risk of bias in each domain. In addition, we graded the quality of evidence of each finding based on the criteria established by the GRADE working group [11]. The quality of study methodology was independently classified by two investigators (YM and HS) as being high, intermediate, low, or very low, based on the study design, risk of bias, indirectness, inconsistency, imprecision, and publication bias. Publication bias was assessed visually using a funnel plot.

### Statistical analysis

We pooled the eligible patients for each outcome and calculated the risk ratios (RRs) and corresponding 95% confidence intervals (CIs) using the Der Simonian-Laird random effects model. Weights were calculated by the inverse variance method for mortality and neurological outcomes, while the mean difference was used for the analyses of decline in ICP and serum sodium levels. We evaluated inter-study heterogeneity using the estimated Cochrane chi-squared test, Tau^2^, and *I*^*2*^ statistics (*I*^*2*^ > 50% indicated severe heterogeneity). We applied unadjusted *p* values to assess significance, with cut-offs for two-tailed *p* values of 0.05 for hypothesis testing and 0.1 for heterogeneity testing. All statistical analyses were performed using Review Manager software (Cochrane systematic review software, version 5.3.5 for Windows; The Nordic Cochrane Centre, the Cochrane Collaboration, Copenhagen, Denmark).

## Results

### Search results

**Fig. 1:**
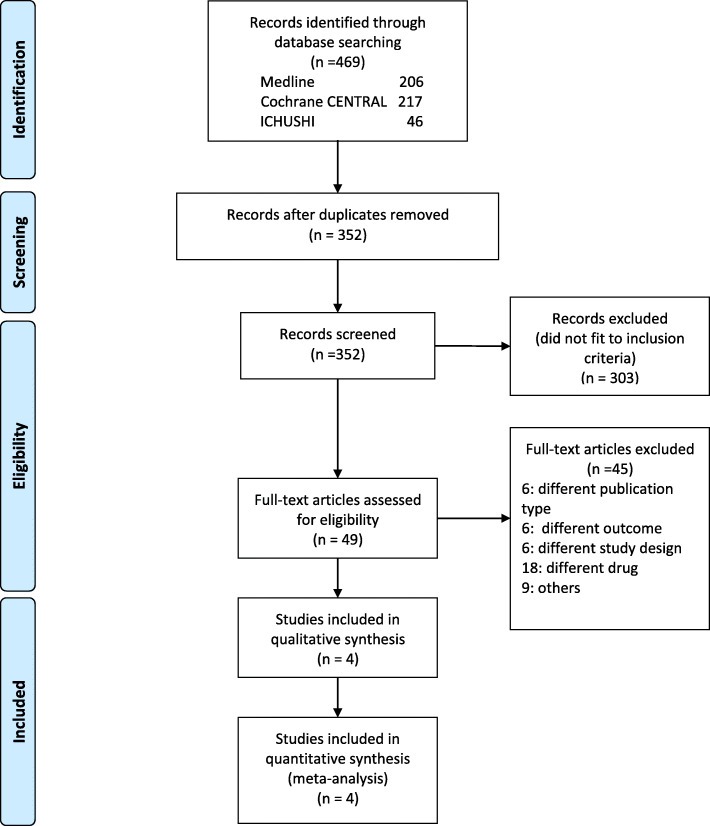
Flow chart for the search strategy and study selection

We identified 352 studies from the electronic databases after eliminating duplicates, although only 49 studies were assessed for eligibility based on the titles and abstracts. After a review of the full-text articles, 45 studies were excluded because of the study design, intervention, outcome, or data only being available in the abstract, despite the corresponding authors being contacted. Thus, four RCTs were included in the meta-analysis (Fig. 1).

### Study characteristics

We analyzed a total of 125 patients from the four RCTs that were reported by Vialet et al. in 2003 [13], Francony et al. in 2008 [14], Cottenceau et al. in 2011 [15], and Jagannatha et al. in 2016 [16] (Table 1). Among 105 patients from three RCTs that evaluated the primary outcome [13, 15, 16], 50 patients were randomly assigned to the HS group and 55 patients were randomly assigned to the mannitol group. The study by Francony et al. was not considered for the primary outcome because it only evaluated ICP reductions. Only one trial had a multicenter design (the study by Vialet et al. [13] included two different intensive care units in two different university hospitals from two different countries). Participants in the study by Francony et al. included some stroke patients (HS group, 2/10 patients [20%]; mannitol group, 1/10 patients [10%]), while the other studies only included TBI patients.

**Table 1:** Baseline characteristics of eligible studies

Study	Type of study	Country	Total number of patients (*n*)	Intervention	Neurological state on admission	Age, years (mean ± SD)	Gender, male/female	Inclusion criteria
Vialet et al. [13]	RCT	France	20	Group 1 (*n* = 10), 7.5% HS, 2 ml/kg; group 2 (*n* = 10), 20% mannitol, 2 ml/kg	Group 1, 4.1 ± 1.6; group 2, 5.4 ± 2.8 (GOS mean ± SD)	Group 1, 35.0 ± 18; group 2, 30.8 ± 19	Group 1, 5/5; group 2, 4/6	TBI patients with informed consent from the closest relative who have persistent coma requiring ICP monitoring and infusion of an osmotic agent to correct refractory episodes of ICP that are resistant to standard modes of therapy
Francony et al. [14]	RCT	France	20	Group 1 (*n* = 10), 7.45% HS, 100 ml; group 2 (*n* = 10), 20% mannitol, 231 ml	Group 1, 7 ± 2; group 2, 8 ± 2 (GCS mean ± SD)	Group 1, 37.0 ± 16; group 2, 43.0 ± 11	Group 1, 9/1; group 2, 7/1	Aged ≥18 years and had sustained elevated ICP of >20 mmHg for > 10 mins, not related to procedural pain.
Cottenceau et al. [15]	RCT	France, Israel	56	Group 1 (*n* = 22), 7.5% HS, 2 ml/kg; group 2 (*n* = 25), 20% mannitol, 4 ml/kg	Group 1, 5 (4–7); group 2, 7 (5–8) (GCS median with lower and upper)	Group 1, 42.7 ± 19.9; group 2, 36.1 ± 16.8	Not available	TBI severe enough to justify ICP monitoring and mechanical ventilation under sedation, with a GCS of ≤ 8 at the time of admission
Jagannatha et al. [16]	RCT	India	38	Group 1 (*n* = 18), 3% HS, 2.5 ml/kg; group 2 (*n* = 20), 20% mannitol, 2.5 ml/kg	Group 1, 4 (4–5); group 2, 5 (4–6) (GCS median with lower and upper)	Group 1, 27.0 ± 8; group 2, 31.0 ± 13	Group 1, 16/2; group 2, 18/2	Patients with severe TBI aged between 15 and 70 years

*RCT* randomized control trial, *HS* hypertonic saline, *GOS* Glasgow outcome scale, *GCS* Glasgow coma scale, *SD* standard deviation, *ICP* intracranial pressure, *TBI* traumatic brain injury, *Group 1* HS group, *Group 2* mannitol group

### Outcomes

**Fig. 2:**
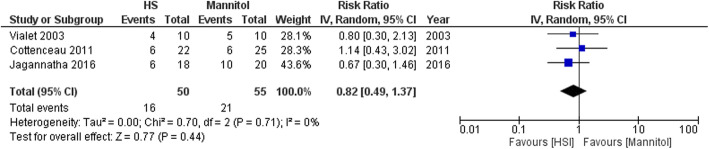
Forest plot comparing the all-cause mortality values between the HS and mannitol groups. HS, hypertonic saline; IV, Inverse variance; CI, confidence interval

**Fig. 3:**
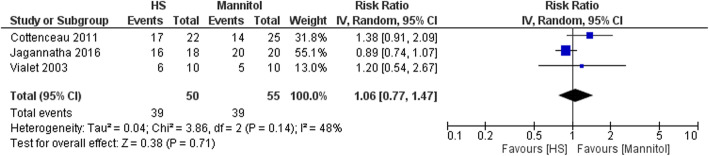
Forest plot comparing the rates of good neurological outcomes between the HS and mannitol groups. HS, hypertonic saline; IV, Inverse variance; CI, confidence interval

**Fig. 4:**
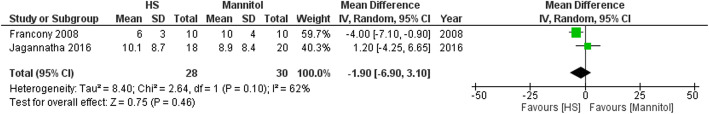
Forest plot comparing the declines in the intracranial pressure between the HS and mannitol groups. HS, hypertonic saline; SD, standard deviation; IV, Inverse variance; CI, confidence interval

**Fig. 5:**
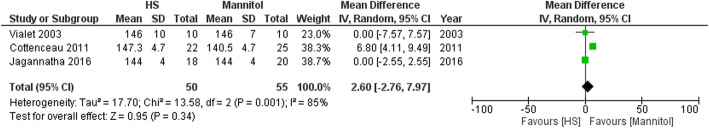
Forest plot comparing the serum sodium levels between the HS and mannitol groups. HS, hypertonic saline; SD, standard deviation; IV, Inverse variance; CI, confidence interval

The forest plot of the primary outcomes is shown in Fig. 2. During the observation period, death was observed for 16 of 50 patients in the HS group (32.0%) and 21 of 55 patients in the mannitol group (38.2%). The difference in risk was not significant between the two infusion strategies (pooled RR, 0.82 [95% CI, 0.49–1.37]) (Fig. 2). The evaluation of 90-day mortality only included two RCTs [13, 16] and the evaluation of 180-day mortality also only included two RCTs [15, 16]. Similar to the result for all-cause mortality, there were no significant differences between the HS and mannitol groups in the 90-day mortality rate (pooled RR, 0.54 [95% CI, 0.23–1.27]) or the 180-day mortality rate (0.82 [95% CI, 0.45–1.52]) (Supplemental file 2). The number of patients with good neurological outcomes tended to be higher in the mannitol group than in the HS group, although the difference was not significant (pooled RR, 1.06 [95% CI, 0.77–1.47]) (Fig. 3). Moreover, there were no significant differences between the groups in the reductions of ICP and serum sodium levels (Figs. 4 and 5).

### Heterogeneity

For the primary outcome (all-cause mortality), no significant heterogeneity was observed among the studies (*I*^*2*^ = 0%, *χ*^2^ = 0.7, *p* = 0.71) (Fig. 2). The heterogeneity evaluations for the other outcomes are described in Supplemental file 2.

### Publication bias, risk of bias, and quality of evidence

**Fig. 6:**
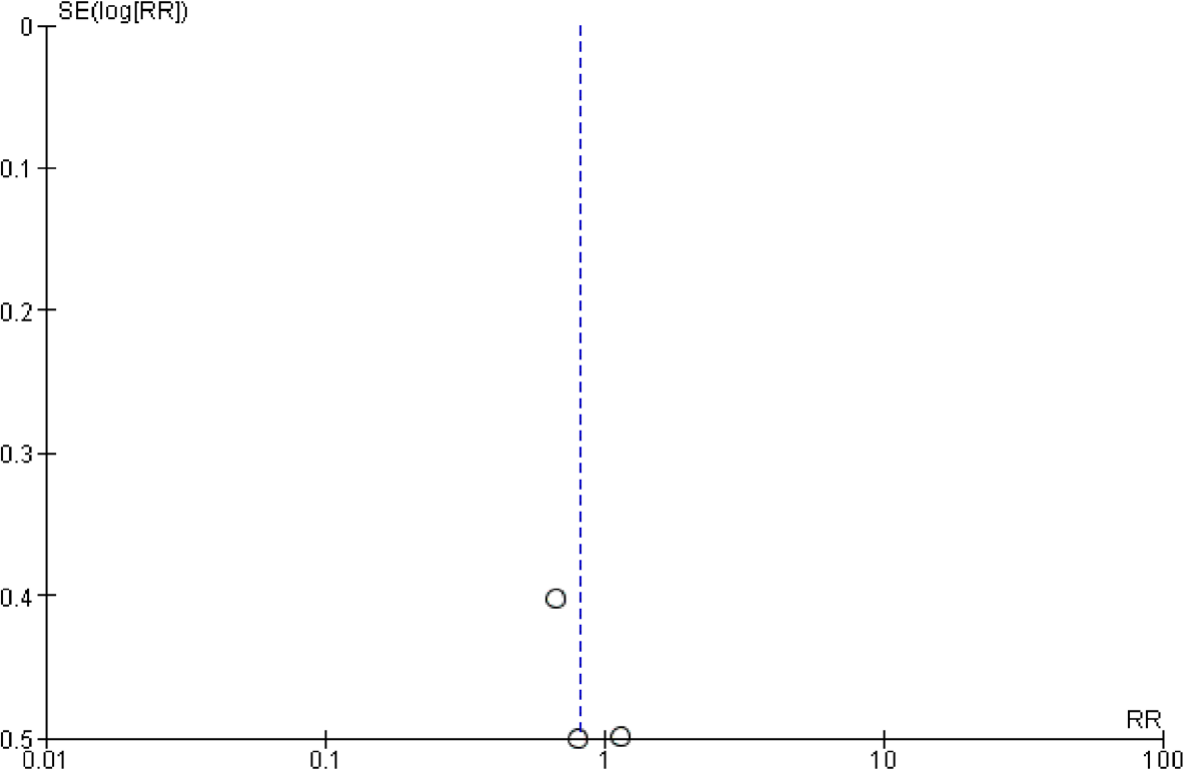
Funnel plot of the three studies included in the meta-analysis of the all-cause mortality. HS, hypertonic saline; SE, standard error; RR, risk ratio

**Fig. 7:**
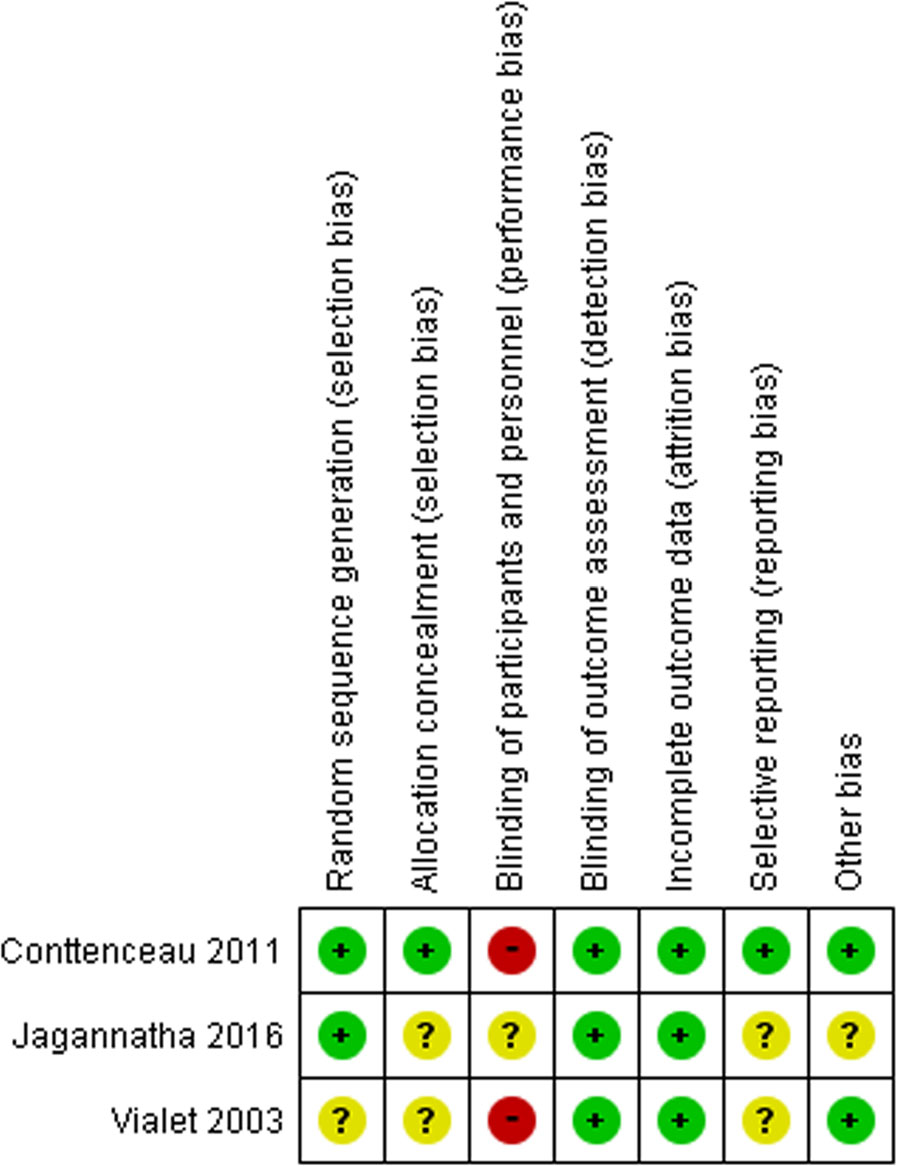
Risk-of-bias summary for the included studies

We also analyzed the presence of publication bias (Fig. 6, Supplemental file 3). A visual inspection of the funnel plot revealed no asymmetry for all-cause mortality. The blinding of participants and personnel was categorized as being associated with a high or an unknown risk of bias in three RCTs due to the nature of the intervention (Fig. 7). The quality of the evidence was rated as very low for the effect of HS on the primary outcomes, with the grade lowered by 3 points due to the risk of biases in blinding and selective reporting, imprecision owing to the small sample sizes, and indirectness due to only ICU settings. The evidence summary is detailed in Table 2.

**Table 2:** Summary of findings

Outcomes	Anticipated absolute effects^*^ (95% CI)		Relative effect (95% CI)	Number of participants (studies)	Certainty of the evidence (GRADE)
	Risk with mannitol	Risk with HS			
All-cause mortality	382 per 1000	**313 per 1000** (187 to 523)	**RR 0.82** (0.49 to 1.37)	105 (3 studies)	Very low
90-days mortality	500 per 1000	**270 per 1000** (115 to 635)	**RR 0.54** (0.23 to 1.27)	58 (2 studies)	Very low
180-days mortality	356 per 1000	**292 per 1000**(160 to 540)	**RR 0.82** (0.45 to 1.52)	85 (2 studies)	Very low
Good neurological outcome	709 per 1000	**752 per 1000** (546 to 1000)	**RR 1.06** (0.77 to 1.47)	105 (3 studies)	Very low
ICP	-	MD **1.9 lower** (6.9 lower to 3.1 higher)	-	58 (2 studies)	Very low
Serum sodium levels	-	MD **2.6 higher** (2.76 lower to 7.97 higher)	-	105 (3 studies)	Very low

*ICP* intracranial pressure, *CI* confidence interval, *HS* hypertonic saline, *MD* mean deviation, RR risk ratio

## Discussion

This study assessed the effects of HS versus mannitol on clinical outcomes in TBI patients. There are few systematic reviews and meta-analyses of RCTs to compare the mortality rates associated with these two strategies [17, 18]. Our meta-analysis revealed that the HS and mannitol strategies were not statistically different in terms of improved clinical outcomes and mortality reductions in TBI patients. However, a large RCT is needed to address this issue, as the included studies had numerous limitations, including differences in the clinical setting at the time of infusion, dosage details, and small sample sizes (Table 1).

In patients with brain injury, ICP is a more powerful predictor of neurological deterioration than cerebral perfusion pressure [19]. Farahvar et al. [20] reported a decrease in the mortality values of patients who responded to ICP-lowering treatment from a large prospectively collected database. Although not entirely patient-centered as an outcome measure, ICP has been used as a prognostic indicator for determining the optimal HS type in some studies. A systematic review revealed that HS and mannitol effectively reduced ICP after TBI [17]. However, a retrospective study of the Brain Trauma Foundation TBI-trac New York State database revealed no significant difference between the HS and mannitol groups in the 2-week mortality rates, although HS was more effective for reducing the cumulative ICP, the daily ICP, and the length of ICU stay [21]. This study also revealed no significant differences between the two groups in terms of mortality and neurological prognosis, which may suggest that HS and mannitol do not have significantly different effects on the clinical outcomes of TBI patients.

Conflicting results were seen between recent meta-analysis [22, 23] and ours. Our results could not show the significant differences of ICP between HS and mannitol whereas two previous meta-analyses [22, 23] showed significant ICP reduction by HS. The current meta-analysis included only two RCTs because we strictly selected eligible studies. We excluded two RCTs [6, 24], which were included in previous meta-analyses; a study reported by Patil did not meet our inclusion criteria and we could not get standard deviation information of ICP [6]. A study reported by Sakellaridis was event based, and the same patients participated in both arms of the treatments [24]. Strict inclusion criteria may become statistically under power. However, if we widely accepted many studies, more studies that are heterogeneous would be included. A guideline suggested using HS over mannitol [25] for the initial management of elevated ICP or cerebral edema for TBI patients although the level of evidence was low. Further studies are necessary to ascertain this claim.

Our results also showed no elevation of sodium between HS and mannitol. Gu et al. reported high sodium levels by HS [23]. They included studies using high concentration of HS (15%), which may result in high sodium levels, whereas our meta-analysis did not. We found relatively low concentration of HS (3–7.5%) might be safely used.

The efficacy of prehospital use of HS is still unclear. ICP is typically measured in the ICU and the evidence regarding the choice of osmotic agent was derived from studies conducted within the ICU setting. A guideline recommended not using HS or mannitol in the prehospital setting to improve neurological outcomes for patients with TBI [25] and we could not include any prehospital studies in this study.

This meta-analysis has several limitations. First, only three RCTs were analyzed for the primary outcome, and those trials had inadequate information regarding ICP changes and small sample sizes. Second, the infusion volumes and concentrations were not uniform across the studies. Third, the participants and healthcare staff were aware of the group assignments in all the included studies, which may have resulted in performance bias. Nevertheless, given the characteristics of the intervention, it would be impossible to conceal the group assignment. Moreover, it is unlikely that this bias would have affected our results, given the use of stratified randomization and objective endpoints. Finally, all included studies were conducted in the ICU setting and included cases without hypovolemia or after hypovolemia normalization. Therefore, well-designed comparative studies are needed to assess these strategies in different situations, such as prehospital resuscitation, and a larger RCT will be required to support our findings.

## Conclusions

Our systematic review and meta-analysis showed no significant difference in the all-cause mortality values associated with HS or mannitol treatment of TBI patients. The certainty of the evidence was considered very low. Current evidence is limited and further studies are warranted to validate our results.

## Supplementary information

**Additional file 1: Supplemental file 1.** Search Strategies in the systematic review.

**Additional file 2: Supplement file 2(a).** Forest plot of the 90-day mortality in comparison between HS and Mannitol group. HS, hypertonic saline; IV, inverse variance. **Supplement file 2(b).** Forest plot of the 180-day mortality in comparison between HS and Mannitol group. HS, hypertonic saline; IV, inverse variance.

**Additional file 3: Supplement file 3(a).** Funnel plot of the 90-day mortality in comparison between HS and Mannitol group. RR, risk ratio. **Supplement file 3(b).** Funnel plot of the 180-day mortality in comparison between HS and Mannitol strategy. RR, risk ratio. Supplement file 3(c). Funnel plot of the good neurological outcome in comparison between HS and Mannitol group. RR, risk ratio. Supplement file 3(d). Funnel plot of the ICP in comparison between HS and Mannitol group. MD, mean difference. Supplement file 3(e). Funnel plot of the serum sodium levels in comparison between HS and Mannitol group. MD, mean difference.

## Data Availability

The data used for this meta-analysis were obtained from the articles corresponding to references [13–16] in our list of references.

## Abbreviations

TBI: Traumatic brain injury
ICP: Intracranial pressure
HS: Hypertonic saline
ICU: Intensive care unit
JRC: The Japan Resuscitation Council
RCT: Randomized controlled trial
GRADE: The Grading of Recommendations Assessment, Development and Evaluation
RR: Risk ratio
CI: Confidence interval
