# Supplementary material for: Effects of hypertonic saline versus mannitol in patients with traumatic brain injury in prehospital, emergency department, and intensive care unit settings: a systematic review and meta-analysis
Source: J Intensive Care. 2020 Aug 12;8:61. doi: 10.1186/s40560-020-00476-x (PMC7425012; doi:10.1186/s40560-020-00476-x)
Supplement: Supplementary file 1 — Additional file 1: Supplemental file 1. Search Strategies in the systematic review. [file 40560_2020_476_MOESM1_ESM.docx]

Supplemental file 1. Search Strategies in the systematic review

1. the MEDLINE database (via PubMed) search strategy（October 1, 2019）

| #1 | “Brain Injuries, Traumatic” [MeSH]) OR (Trauma* [tiab] AND Brain [tiab] AND Injur* [tiab]) OR TBI [tiab] | 53256 |
| --- | --- | --- |
| #2 | "Craniocerebral Trauma" [Mesh] OR "Craniocerebral Trauma" [tiab] | 153428 |
| #3 | “Cerebrovascular Trauma” [Mesh] OR “Cerebrovascular Trauma” [tiab] | 7019 |
| #4 | “Intracranial Hypertension” [Mesh] OR “Intracranial Hypertension” [tiab] | 13379 |
| #5 | “Brain edema” [Mesh] OR “Brain edema” [tiab] OR “Brain oedema” [tiab] | 17631 |
| #6 | #1 OR #2 OR #3 OR #4 OR #5 | 201260 |
| #7 | “Saline Solution, Hypertonic” [Mesh] OR “Hypertonic Solutions” [Mesh] OR (Hypertonic [tiab] AND Solution* [tiab]) | 14098 |
| #8 | “Diuretics, Osmotic” [Mesh] OR (Osmotic [tiab] AND Diuretic* [tiab]) | 1448 |
| #9 | Mannitol [Mesh] OR Mannitol [tiab] | 22863 |
| #10 | Glycerol [Mesh] OR Glycerol [tiab] OR Glyceol [tiab] | 56597 |
| #11 | Isosorbide [Mesh] OR Isosorbide [tiab] | 4772 |
| #12 | #7 OR #8 OR #9 OR #10 OR #11 | 96389 |
| #13 | randomized controlled trial[pt] OR controlled clinical trial[pt] OR randomized[tiab] OR placebo[tiab] OR clinical trials as topic[mesh:noexp] OR randomly[tiab] OR trial[ti] NOT (animals[mh] NOT humans [mh]) | 1150091 |
| #14 | #6 AND #12 AND #13 | 206 |

2. the Cochrane Central Register of Controlled Trials search strategy

| #1 | MeSH descriptor: [Brain Injuries, Traumatic] explode all trees | 569 |
| --- | --- | --- |
| #2 | (Trauma*):ti,ab,kw | 23119 |
| #3 | (Brain):ti,ab,kw | 55728 |
| #4 | (Injur*):ti,ab,kw | 55099 |
| #5 | #2 AND #3 AND #4 | 3974 |
| #6 | (TBI):ti,ab,kw | 2520 |
| #7 | MeSH descriptor: [Craniocerebral Trauma] explode all trees | 3189 |
| #8 | ("Craniocerebral Trauma"):ti,ab,kw | 348 |
| #9 | MeSH descriptor: [Cerebrovascular Trauma] explode all trees | 32 |
| #10 | (“Cerebrovascular Trauma”):ti,ab,kw | 5 |
| #11 | MeSH descriptor: [Intracranial Hypertension] explode all trees | 184 |
| #12 | (“Intracranial Hypertension”):ti,ab,kw | 613 |
| #13 | MeSH descriptor: [Brain Edema] explode all trees | 189 |
| #14 | (“Brain edema”):ti,ab,kw | 615 |
| #15 | (“Brain oedema”):ti,ab,kw | 615 |
| #16 | #1 OR #5 OR #6 OR #7 OR #8 OR #9 OR #10 OR #11 OR #12 OR #13 OR #14 OR #15 | 7232 |
| #17 | MeSH descriptor: [Saline Solution, Hypertonic] explode all trees | 495 |
| #18 | MeSH descriptor: [Hypertonic Solutions] explode all trees | 688 |
| #19 | (Solution*):ti,ab,kw | 1983 |
| #20 | (imipenem):ti,ab,kw | 47849 |
| #21 | #19 AND #20 | 1090 |
| #22 | MeSH descriptor: [Diuretics, Osmotic] explode all trees | 80 |
| #23 | (Osmotic):ti,ab,kw | 1343 |
| #24 | (Diuretic*):ti,ab,kw | 8905 |
| #25 | #23 AND #24 | 132 |
| #26 | MeSH descriptor: [Mannitol] explode all trees | 572 |
| #27 | (Mannitol):ti,ab,kw | 1653 |
| #28 | MeSH descriptor: [Glycerol] explode all trees | 765 |
| #29 | (Glycerol):ti,ab,kw | 1861 |
| #30 | (Glyceol):ti,ab,kw | 3 |
| #31 | MeSH descriptor: [Isosorbide] explode all trees | 952 |
| #32 | (Isosorbide):ti,ab,kw | 1784 |
| #33 | #17 OR #18 OR #21 OR #22 OR #25 OR #26 OR #27 OR #28 OR #29 OR #30 OR #31 OR #32 | 6308 |
| #34 | #16 AND #33 | 217 |

3. the Igaku Chuo Zasshi (ICHUSHI; Japanese) database search strategy

| #1 | (Brain Injuries, Traumatic/TH (Thesaurus) or Brain Injuries, Traumatic/TA (Title and Abstract) or (Traumatic/TA and Brain Injuries/TA) or TBI/TA) and (Publication Type (PT)=Excluding proceedings) | 4115 |
| --- | --- | --- |
| #2 | Head injury (Head injury/TH or Head injury/TA) and (PT= Excluding proceedings) | 25914 |
| #3 | (Cerebrovascular trauma/TH or Cerebrovascular trauma/TA) and (PT=Excluding proceedings) | 1505 |
| #4 | (Increased intracranial pressure/TH or Increased intracranial pressure /TA) and (PT=Excluding proceedings) | 3210 |
| #5 | (Brain edema /TH or Brain edema /TA) and (PT= Excluding proceedings) | 3834 |
| #6 | #1 or #2 or #3 or #4 or #5 | 33923 |
| #7 | (Hypertonic saline/TH or Hypertonic saline/TA) and (PT=Excluding proceedings) | 801 |
| #8 | (Hypertonic solution/TH or Hypertonic solution/TA) and (PT= Excluding proceedings) | 861 |
| #9 | (Osmotic diuretics/TH or Osmotic diuretics/TA) and (PT= Excluding proceedings) | 1427 |
| #10 | (Mannitol/TH or Mannitol/TA) and (PT= Excluding proceedings) | 1524 |
| #11 | (Glycerol/TH or Glycerol/TA or Glycerol/TA) and (PT= Excluding proceedings) | 4195 |
| #12 | (Isosorbide/TH or Isosorbide/TA) and (PT= Excluding proceedings) | 1683 |
| #13 | #7 or #8 or #9 or #10 or #11 or #12 | 8323 |
| #14 | #6 and #13 | 690 |
| #15 | Randomized controlled trial/TH or Quasi-randomized controlled trial /TH or Randomization/AL (All Field) or Controlled trial/AL or Clinical trial/AL or placebo/AL or Control/AL or Clinical trial/AL | 308132 |
| #16 | #14 and #15 | 46 |

*These search terms are originally written in Japanese.
