# Supplementary material for: Effects of hypertonic saline versus mannitol in patients with traumatic brain injury in prehospital, emergency department, and intensive care unit settings: a systematic review and meta-analysis
Source: J Intensive Care. 2020 Aug 12;8:61. doi: 10.1186/s40560-020-00476-x (PMC7425012; doi:10.1186/s40560-020-00476-x)
Supplement: Supplementary file 2 — Additional file 2: Supplement file 2(a). Forest plot of the 90-day mortality in comparison between HS and Mannitol group. HS, hypertonic saline; IV, inverse variance. Supplement file 2(b). Forest plot of the 180-day mortality in comparison between HS and Mannitol group. HS, hypertonic saline; IV, inverse variance. [file 40560_2020_476_MOESM2_ESM.docx]

Supplement file 2(a)


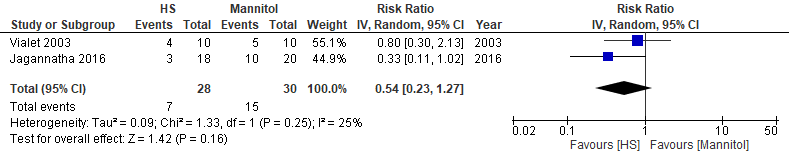


Forest plot of the 90-day mortality in comparison between HS and Mannitol group.

HS, hypertonic saline; IV, inverse variance

Supplement file 2(b)


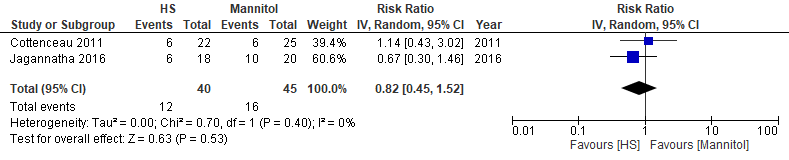


Forest plot of the 180-day mortality in comparison between HS and Mannitol group.

HS, hypertonic saline; IV, inverse variance
