# Supplementary material for: Effects of hypertonic saline versus mannitol in patients with traumatic brain injury in prehospital, emergency department, and intensive care unit settings: a systematic review and meta-analysis
Source: J Intensive Care. 2020 Aug 12;8:61. doi: 10.1186/s40560-020-00476-x (PMC7425012; doi:10.1186/s40560-020-00476-x)
Supplement: Supplementary file 3 — Additional file 3: Supplement file 3(a). Funnel plot of the 90-day mortality in comparison between HS and Mannitol group. RR, risk ratio. Supplement file 3(b). Funnel plot of the 180-day mortality in comparison between HS and Mannitol strategy. RR, risk ratio. Supplement file 3(c). Funnel plot of the good neurological outcome in comparison between HS and Mannitol group. RR, risk ratio. Supplement file 3(d). Funnel plot of the ICP in comparison between HS and Mannitol group. MD, mean difference. Supplement file 3(e). Funnel plot of the serum sodium levels in comparison between HS and Mannitol group. MD, mean difference. [file 40560_2020_476_MOESM3_ESM.docx]

Supplement file 3(a)


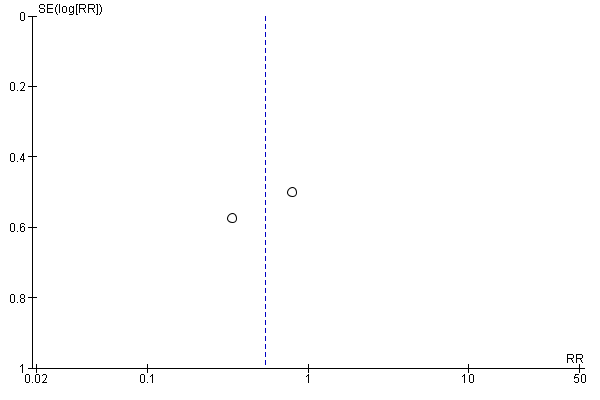


Funnel plot of the 90-day mortality in comparison between HS and Mannitol group.

RR, risk ratio

Supplement file 3(b)


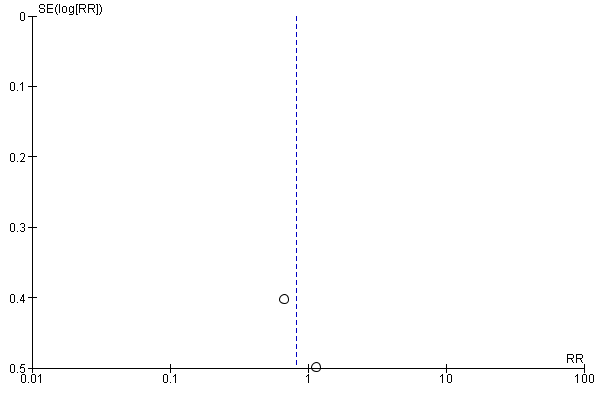


Funnel plot of the 180-day mortality in comparison between HS and Mannitol strategy.

RR, risk ratio

Supplement file 3(c)


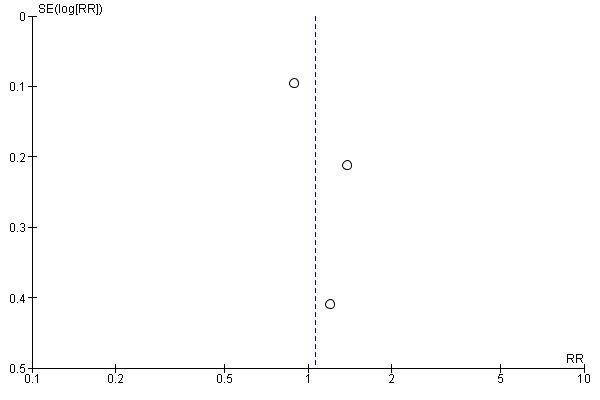


Funnel plot of the good neurological outcome in comparison between HS and Mannitol group.

RR, risk ratio

Supplement file 3(d)


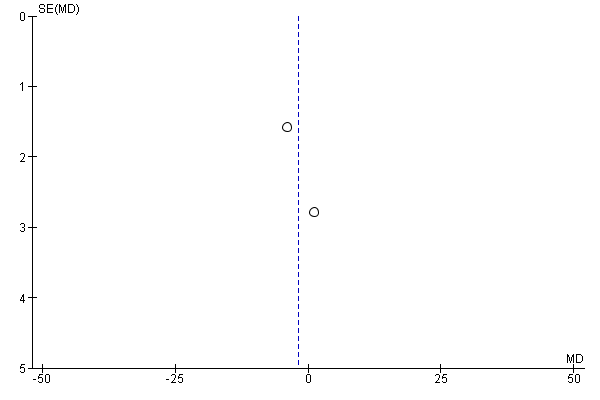


Funnel plot of the ICP in comparison between HS and Mannitol group.

MD, mean difference

Supplement file 3(e)


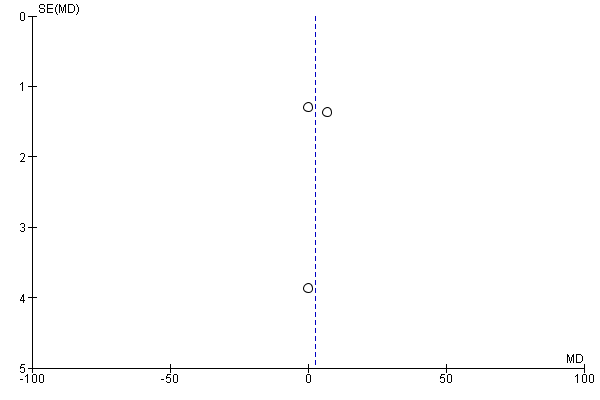


Funnel plot of the serum sodium levels in comparison between HS and Mannitol group.

MD, mean difference
